# Editorial: Ion Channel Trafficking and Cardiac Arrhythmias

**DOI:** 10.3389/fphys.2018.01254

**Published:** 2018-09-25

**Authors:** Marcel A. G. van der Heyden, Brian P. Delisle, Hugues Abriel

**Affiliations:** ^1^Division of Heart and Lungs, Department of Medical Physiology, University Medical Center Utrecht, Utrecht, Netherlands; ^2^Department of Physiology, University of Kentucky, Lexington, KY, United States; ^3^Institute of Biochemistry and Molecular Medicine, Swiss National Centre of Competence in Research TransCure, University of Bern, Bern, Switzerland

**Keywords:** ion channel trafficking, protein complexes, Kv11.1, Nav1.5, Kir2.1, connexin 43, TRPM4, autophagy

The cardiac action potential is one of the most exquisite examples of regulated protein expression that nature presents. The balanced expression of many underlying channel proteins that transport ions with great selectivity at the sarcolemma of cardiomyocytes is crucial to maintain regular cardiac contraction throughout one's lifetime. The coordinated expression must display both robustness and flexibility to sustain stability in action potential generation during homeostasis and rapid adaptation in response to altered physiological demands. Moreover, regional differences in action potential shape within the heart allude to the tuning and fine tuning of functional ion channel expression at the sarcolemma.

The cellular processes that transport channel proteins from the endoplasmic reticulum toward specified regions on the sarcolemma and, subsequently, remove them from the plasma membrane and direct them to the protein degradation machinery are commonly known as trafficking. Recycling and additional intracellular transport mechanisms are contained within this definition. Importantly, several forms of congenital long QT syndrome result from the aberrant trafficking of ion channels. For example, many mutations in the *KCNH2* gene, encoding the Kv11.1 (hERG) potassium channel, result in incomplete glycosylation and decreased levels of functional expression at the sarcolemma (Anderson et al., 2014). In addition, drugs that induce arrhythmia also disturb normal trafficking and, hence, alter cardiac electrophysiology (de Git et al., 2013). Finally, cardiac remodeling processes that are associated with cardiac disease also affect the trafficking of ion channels and gap junction proteins (Basheer and Shaw, 2016).

Research on ion channel trafficking is complex as it requires a multidisciplinary approach that combines a plethora of techniques that include molecular biology and cell biology, biochemistry, imaging, and electrophysiology to name a few. Furthermore, the field of cardiac ion channel trafficking is challenged to apply new concepts from basic cell biology that focus on the general structure and basic mechanisms of intracellular transport toward the level of individual ion channels.

It is the purpose of this Frontiers Research Topic on ion channel trafficking and cardiac arrhythmias to reveal some new levels of complexity and to implicitly provide new directions that the field must explore.

Balse and Boycot provide a review on the basic mechanisms of ion channel trafficking, their interdependence on basal cellular (e.g., local lipid composition) and extracellular (e.g., potassium concentration) factors, and some examples of modifying ion channel trafficking in order to counteract arrhythmias. The role of anchoring proteins in steering channel trafficking and membrane localization and their involvement in human cardiac disease is described in the review by El Refaey and Mohler. Thereafter, the role of non-pore-forming Nav-β-subunits channel proteins in trafficking of sodium and also the potassium channels and their importance in normal channel distribution within the cardiomyocyte are discussed by Edokobi and Isom. In their perspective paper, Van Bavel et al. emphasize on the involvement of autophagy pathways in cardiac ion channel trafficking under disease conditions and the impact of drugs, including antiarrhythmics, on autophagy and subsequent arrhythmic outcomes.

The development of a single cell high-throughput system to detect aberrant Kv11.1 trafficking is the aim of the original contribution by Kanner et al. An important implication of their findings is that full glycosylation of the Kv11.1 protein is not, by definition, equal to plasma membrane localization. Hall et al. provide compelling evidence for mutation specific differences in Kv11.1 trafficking defects, that is, variations in intracellular retention, which may have consequences for future treatment options. Importantly, they also demonstrate that the findings obtained in HEK293 cells can be replicated in cardiomyocytes derived from human induced pluripotent stem cells.

Ion channel interdependence with respect to expression and function is the focus of the original contributions by Utrilla et al. and Portero et al. The trafficking properties of the Kir2.1-Nav1.5 channel complex are different from those of the individual channels. Utrilla et al. found the dominance of Nav1.5 over Kir2.1 with respect to regulation of internalization, CamKII sensitivity, and degradation. Portero et al. demonstrate the impact of Kv4.3 channel expression on the Nav1.5 current in an ectopic expression system and how this may relate to gain-of-function mutations in the Kv4.3 coding gene in Brugada syndrome. Both studies demonstrate the importance of studying the trafficking of a single channel in the context of other channel types. In view of the balanced channel expression required to generate a cardiac action potential, such channel complex formation and interdependence may not come as a surprise.

Bianchi et al. describe new mutations in TRPM4 channels that are associated with complete heart block or idiopathic ventricular fibrillation and found that the gain- and loss-of-expression is directly related to differences in protein stability in the plasma membrane. Taneja et al. build upon their earlier work on Kir2.1 anterograde transport (Li et al., 2016) and provide evidence that the Golgi-tether Golgin-97 is involved in Kir2.1 transport through the Golgi apparatus to reach the trans-Golgi-network.

Finally, Fu et al. identified an additional function of the connexin 43 isoform GJA1-20k. This connexin 43 isoform increases in expression upon cellular stress, promotes mitochondrial transport along the microtubules, and preserves normal cellular localization of the mitochondria. This study also implicates that ion channel proteins can have additional “noncanonical” cellular functions apart from ion conduction across the plasma membrane. From a physiological point of view, we anticipate that adaptations in action potential features and expression of their underlying ion channels may have direct impact on a number of cellular processes and signaling mechanisms that are initiated by the channel proteins themselves.

Several lessons can be taken from this Frontiers Research Topic. (1) Ion channel trafficking is of great complexity in which each channel protein type follows its own specific path, and even different mutations within the same protein can affect different steps. (2) Ion channel complexes exhibit different trafficking properties when compared to their individual components. (3) Ectopic expression of one channel protein type reveals intrinsic trafficking properties that can be reproduced in cardiac cell types, but full appreciation of trafficking behavior in view of action potential properties may require co-expression of additional channel types. (4) Channel proteins are versatile and have additional cellular functions, apart from conducting ions across the plasma membrane, in intracellular signaling and in regulating subcellular architecture.

Finally, the field of cardiac ion channel trafficking is progressing rapidly. As indicated by several authors in this Frontiers special issue, now the field has to progress from ectopic cell systems toward adult cardiomyocytes. Furthermore, we encourage the study of channel trafficking *in vivo* to unequivocally determine the direct link between aberrant ion channel trafficking and cardiac arrhythmia.

## Author contributions

MH drafted and planned the editorial. HA drafted and rewrote the manuscript. BD reread, corrected, and reworded the manuscript.

### Conflict of interest statement

The authors declare that the research was conducted in the absence of any commercial or financial relationships that could be construed as a potential conflict of interest.
